# Supplementary material for: Do non-traumatic stressful life events and ageing negatively impact working memory performance and do they interact to further impair working memory performance?
Source: PLoS One. 2023 Nov 29;18(11):e0290635. doi: 10.1371/journal.pone.0290635 (PMC10686508; doi:10.1371/journal.pone.0290635)
Supplement: S1 Table — (PDF) [file pone.0290635.s001.pdf]

S2 Table. Age Median and IQR values for Young and Older Adult participants by study

	Median (IQR)	
**Studies**	Young Adults	Older Adults
Study 1	19 (19-22)	69 (64-73)
Study 2A	29 (25-32)	63 (61-67)
Study 2B	29 (24-31.5)	64 (61-65)
