# Supplementary material for: Do non-traumatic stressful life events and ageing negatively impact working memory performance and do they interact to further impair working memory performance?
Source: PLoS One. 2023 Nov 29;18(11):e0290635. doi: 10.1371/journal.pone.0290635 (PMC10686508; doi:10.1371/journal.pone.0290635)
Supplement: S2 Table — (PDF) [file pone.0290635.s002.pdf]

S4 Table. Percent correct and d-prime means, standard errors and univariate ANOVA F values by age group and stress group for the 3 replication studies.

				Univariate ANOVA F-values		
		**Young Adults**	**Older Adults**	**YA vs. OA**	**Low Stress vs. High Stress**	**Age Grp x Stress Grp**
	**Accuracy (% correct)**	mean (SE)	mean (SE)	F	F	F
**Study 1**	*Low Stress (n=21)*	93.71 (0.81)	89.00 (1.98)	3.579	6.295**	0.026
	High Stress (n=19)	87.58 (3.21)	83.61 (2.79)			
**Study 2A**	*Low Stress (n=30)*	91.77 (1.78)	91.13 (1.37)	0.393	3.309	0.105
	High Stress (n=28)	88.61 (2.53)	86.60 (2.60)			
**Study 2B**	*Low Stress (n=30)*	90.72 (2.50)	89.83 (2.01)	0.963	0.309	1.960
	High Stress (n=28)	88.93 (2.52)	93.99 (0.88)			

	**Accuracy (d-prime)**	mean (SE)	mean (SE)	F	F	F
**Study 1**	*Low Stress (n=21)*	3.06 (0.11)	2.56 (0.2)	5.514*	4.990*	0.000
	High Stress (n=19)	2.58 (0.27)	2.07 (0.28)			
**Study 2A**	*Low Stress (n=30)*	2.96 (0.17)	2.79 (0.16)	1.290	2.790	0.102
	High Stress (n=28)	2.68 (0.23)	2.38 (0.26)			
**Study 2B**	*Low Stress (n=30)*	2.89 (0.21)	2.69 (0.19)	0.467	0.252	2.980
	High Stress (n=28)	2.66 (0.23)	3.11 (0.11)			

* significant at < 0.05 (two-tailed).

** significant at < 0.01 (two-tailed).
