# Supplementary material for: Do non-traumatic stressful life events and ageing negatively impact working memory performance and do they interact to further impair working memory performance?
Source: PLoS One. 2023 Nov 29;18(11):e0290635. doi: 10.1371/journal.pone.0290635 (PMC10686508; doi:10.1371/journal.pone.0290635)
Supplement: S4 Table — (PDF) [file pone.0290635.s004.pdf]

**S8 Table. Median split value with inter-quartile range by study and combined.**

| | | LESS (n=81) | SRRS (n=75) |
|---|---|---|---|
| Study | n | **median (IQR)** | **median (IQR)** |
| Study 1 | 40 | 592 (492.00 - 639.50) | 913 (753.00 - 1009.00) |
| Study 2A | 58 | 577 (357.00 - 691.00) | 738 (632.00 - 845.00) |
| Study 2B | 58 | 516 (348.00 - 692.00) | 766 (684.50 - 849.50) |
| **Study 1, 2A, 2B combined** | **156** | **577 (383.50 - 660.00)** | **786 (685.00 - 873.00)** |
