# Supplementary material for: Do non-traumatic stressful life events and ageing negatively impact working memory performance and do they interact to further impair working memory performance?
Source: PLoS One. 2023 Nov 29;18(11):e0290635. doi: 10.1371/journal.pone.0290635 (PMC10686508; doi:10.1371/journal.pone.0290635)
Supplement: S5 Table — (PDF) [file pone.0290635.s005.pdf]

S9 Table. Sensitivity analysis: A comparison using a single median split.

Table 1. Means, standard errors and Univariate ANOVA F values by age group and stress group for the 3 replication studies.

				Univariate ANOVA F-values		
		**Young Adults**	**Older Adults**	YA vs. OA	Low Stress vs. High Stress	Age Grp x Stress Grp
	Accuracy (% correct)	*mean (SE)*	*mean (SE)*	F	F	F
**Study 1**	*Low Stress (n=13)*	93.96 (0.98)	87.83 (4.06)	2.976	1.789	0.382
	High Stress (n = 27)	88.85 (2.53)	85.95 (1.98)			
**Study 2A**	*Low Stress (n=33)*	91.77 (1.77)	90.44 (1.55)	0.663	2.756	0.040
	High Stress (n = 25)	88.61 (2.41)	86.42 (3.04)			
**Study 2B**	*Low Stress (n=33)*	91.27 (2.26)	90.42 (1.91)	1.299	0.004	2.371
	High Stress (n = 25)	87.85 (2.89)	93.59 (0.84)			
	Reaction time (ms)					
**Study 1**	*Low Stress (n=13)*	698.43 (46.00)	804.99 (45.19)	2.953	8.423**	0.061
	High Stress (n = 27)	890.76 (83.57)	1033.24 (46.72)			
**Study 2A**	*Low Stress (n=33)*	698.19 (67.02)	726.37 (52.30)	2.088	0.866	0.998
	High Stress (n = 25)	576.26 (48.36)	730.71 (87.81)			
**Study 2B**	*Low Stress (n=33)*	712.57 (43.71)	780.15 (52.79)	2.510	0.122	0.041
	High Stress (n = 25)	685.55 (51.98)	772.97 (43.96)			

* significant at < 0.05 (two-tailed)
** significant at < 0.01 (two-tailed)

Table 2. Age group percent correct and RT mean differences, standard errors and Bayes Factors for all studies using a single median split.

		Young vs. Older Adults				
Accuracy (% correct)		***Prior***	***Likelihood***	***Posterior***		
	N: Incremental Increase	*mean Difference (SE)*	*mean Difference (SE)*	*mean Difference (SE)*	95% credible interval[a]	BF
**Marshall et al. (N=60)**	**60**	5.00[a]	4.83 (1.64)			15.99[†]
**Study 1 (N=40)**	**100**	4.83 (1.64)	4.35 (2.42)	4.68 (1.36)	2.02, 7.34	1.59
**Study 2A (N=58)**	**158**	4.68 (1.36)	1.29 (2.12)	3.70 (1.14)	1.46, 5.94	0.58
**Study 2B (N=58)**	**216**	3.70 (1.14)	-1.98 (2.06)	2.37 (1.00)	0.41, 4.33	0.78
Reaction Time (ms)			*mean Difference (SE)*		95% credible interval[a]	BF
**Marshall et al. (N=46)**	**46**	50.00[a]	-441.28 (109.07)			3.77[†]
**Study 1 (N=40)**	**86**	-441.28 (109.07)	-155.69 (71.44)	-241.43 (59.76)	-358.56, -124.30	2.64
**Study 2A (N=58)**	**144**	-241.43 (59.76)	-88.79 (60.04)	-165.46 (42.35)	-248.47, -82.44	1.22
**Study 2B (N=58)**	**202**	-165.46 (42.35)	-75.54 (46.79)	-124.96 (31.40)	-186.50, -63.42	1.33

[a] In the first iteration, an estimated maximum performance difference of 10% was assumed. Half of this value was used as a vague prior. This prior was used to calculate the BF for Marshall et al. (2015)'s result.

[†] evidence favours H1

[‡] evidence favours H0

Table 3. Cumulative stress percent correct and RT mean differences, standard errors and Bayes Factors for all studies using a single median split.

		**Low Stress vs. High Stress**				
Accuracy (% correct)		***Prior***	***Likelihood***	***Posterior***		
	N: Incremental Increase	*mean Difference (SE)*	*mean Difference (SE)*	*mean Difference (SE)*	95% credible interval[a]	BF
**Marshall et al. (N=60)**	**60**	5.00[a]	3.50 (1.62)			2.54
**Study 1 (N=40)**	**100**	3.5 (1.62)	4.26 (2.35)	3.74 (1.33)	1.13, 6.36	1.44
**Study 2A (N=58)**	**158**	3.74 (1.33)	3.35 (2.24)	3.64 (1.15)	1.39, 5.89	1.22
**Study 2B (N=58)**	**216**	3.64 (1.15)	0.03 (2.16)	2.85 (1.01)	0.86, 4.83	0.59
Reaction Time (ms)		***mean Difference (SE)***	*mean Difference (SE)*	*mean Difference (SE)*	95% credible interval[a]	BF
**Marshall et al. (N=46)**	**46**	50.00[a]	62.33 (158.09)			0.96
**Study 1 (N=40)**	**86**	62.33 (158.09)	-225.23 (62.27)	-186.6 (57.94)	-300.16, -73.04	3.32[†]
**Study 2A (N=58)**	**144**	-186.6 (57.94)	74.67 (63.98)	-68.88 (42.95)	-153.06, 15.29	1.03
**Study 2B (N=58)**	**202**	-68.88 (42.95)	14.32 (46.76)	-30.81 (31.63)	-92.8, 31.19	0.99

[a] In the first iteration, an estimated maximum performance difference of 10% was assumed. Half of this value was used as a vague prior. This prior was used to calculate the BF for Marshall et al. (2015)'s result.

[†] evidence favours H1

[‡] evidence favours H0

Table 4. Percent correct and RT mean differences, standard errors and Bayes Factors for young low and high stress groups by older low and high stress groups interaction effect for all studies using a single median split.

		Age by Stress Group Interaction				
		Prior	***Likelihood***	***Posterior***		
Accuracy (% correct)	N: Incremental Increase	*mean Difference (SE)*	*mean Difference (SE)*	*mean Difference (SE)*	95% credible interval[a]	BF
**Marshall et al. (N=60)**	**60**	2.50[a]	-12.55 (2.85)			50.86[†]
**Study 1 (N=40)**	**100**	-12.55 (2.85)	4.23 (4.73)	-8.08 (2.44)	-12.86, -3.29	0.78
**Study 2A (N=58)**	**158**	-8.08 (2.44)	-0.93 (4.36)	-6.37 (2.13)	-10.55, -2.20	0.94
**Study 2B (N=58)**	**216**	-6.37 (2.13)	7.26 (4.22)	-3.60 (1.90)	-7.33, 0.13	1.06
Reaction Time (ms)	***N: Incremental Increase***	***mean Difference (SE)***	*mean Difference (SE)*	*mean Difference (SE)*	95% credible interval[a]	BF
**Marshall et al. (N=46)**	**46**	25.50[a]	-47.75 (254.72)			1.00
**Study 1 (N=40)**	**86**	-47.75 (254.72)	83.49 (119.97)	59.66 (108.53)	-153.07, 272.39	0.99
**Study 2A (N=58)**	**144**	59.66 (108.53)	64.79 (125.86)	61.85 (82.19)	-99.25, 222.95	0.70
**Study 2B (N=58)**	**202**	61.85 (82.19)	48.72 (95.88)	56.29 (62.40)	-66.02, 178.60	0.68

[a] In the first iteration, an estimated maximum performance difference of 10% was assumed. Half of this value was used as a vague prior. This prior was used to calculate the BF for Marshall et al. (2015)'s result.

[†] evidence favours H1

[‡] evidence favours H0

Table 5a.YA percent correct and RT mean differences, standard errors and Bayes Factors by stress group within age group for all studies using a single median split.

		YA: Low vs. High Stress				
		Prior	***Likelihood***	***Posterior***		
Accuracy (% correct)	N: Incremental Increase	*mean Difference (SE)*	*mean Difference (SE)*	*mean Difference (SE)*	95% credible Interval[a]	BF
**Marshall et al. (N=60)**	**60**	5.00[a]	-2.39 (1.88)			0.71
**Study 1 (N=40)**	**100**	-2.39 (1.88)	5.11 (2.70)	0.05 (1.54)	-2.97, 3.08	1.23
**Study 2A (N=58)**	**158**	0.05 (1.54)	3.16 (2.93)	0.73 (1.36)	-1.95, 3.40	1.00
**Study 2B (N=58)**	**216**	0.73 (1.36)	3.43 (3.58)	1.07 (1.27)	-1.43, 3.57	0.98
Reaction Time (ms)	***Sample Size Increase***	***mean Difference (SE)***	*mean Difference (SE)*	*mean Difference (SE)*	95% credible Interval[a]	BF
**Marshall et al. (N=46)**	**46**	12.50[a]	-87.32 (78.59)			1
**Study 1 (N=40)**	**86**	-87.32 (78.59)	-192.33 (93.47)	-130.81 (60.16)	-248.71, -12.91	1.32
**Study 2A (N=58)**	**144**	-130.81 (60.16)	121.94 (81.12)	-41.14 (48.32)	-135.84, 53.57	1.00
**Study 2B (N=58)**	**202**	-41.14 (48.32)	27.01 (68.67)	-18.57 (39.52)	-96.02, 58.88	0.81

[a] In the first iteration, an estimated maximum performance difference of 10% was assumed. Half of this value was used as a vague prior. This prior was used to calculate the BF for Marshall et al. (2015)'s result.

† evidence favours H1 (> 3)

‡ evidence favours H0 (< ⅓)

Table 5b. OA percent correct and RT mean differences, standard errors and Bayes Factors by stress group within age group for all studies using a single median split.

		OA: Low vs. High Stress				
		Prior	***Likelihood***	***Posterior***		
Accuracy (% correct)	N: Incremental Increase	*mean Difference (SE)*	*mean Difference (SE)*	*mean Difference (SE)*	95% credible Interval[a]	BF
**Marshall et al. (N=60)**	**60**	12.50[a]	9.39 (2.01)			>100[†]
**Study 1 (N=40)**	**100**	9.39 (2.01)	1.88 (4.49)	8.13 (1.84)	4.53, 11.74	0.72
**Study 2A (N=58)**	**158**	8.13 (1.84)	4.02 (3.34)	7.18 (1.61)	4.02, 10.34	0.86
**Study 2B (N=58)**	**216**	7.18 (1.61)	-3.17 (2.09)	3.33 (1.28)	0.83, 5.83	1.01
Reaction Time (ms)	***Sample Size Increase***	***mean Difference (SE)***	*mean Difference (SE)*	*mean Difference (SE)*	95% credible Interval[a]	BF
**Marshall et al. (N=46)**	**46**	37.50[a]	102.87 (209.13)			0.99
**Study 1 (N=40)**	**86**	102.87 (209.13)	-228.25 (64.86)	-199.20 (61.95)	-320.62, -77.79	8.56[†]
**Study 2A (N=58)**	**144**	-199.20 (61.95)	-4.33 (100.57)	-145.60 (52.74)	-248.98, -42.22	0.79
**Study 2B (N=58)**	**202**	-145.60 (52.74)	7.18 (66.46)	-86.56 (41.31)	-167.54, -5.59	0.85

[a] In the first iteration, an estimated maximum performance difference of 10% was assumed. Half of this value was used as a vague prior. This prior was used to calculate the BF for Marshall et al. (2015)'s result.

[†] evidence favours H1 (> 3)

[‡] evidence favours H0 (< ⅓)
