# Supplementary material for: Do non-traumatic stressful life events and ageing negatively impact working memory performance and do they interact to further impair working memory performance?
Source: PLoS One. 2023 Nov 29;18(11):e0290635. doi: 10.1371/journal.pone.0290635 (PMC10686508; doi:10.1371/journal.pone.0290635)
Supplement: S1 Appendix — (PDF) [file pone.0290635.s006.pdf]

S1 Appendix 1

Study 1 Participant record (in-person study, part of wider project)

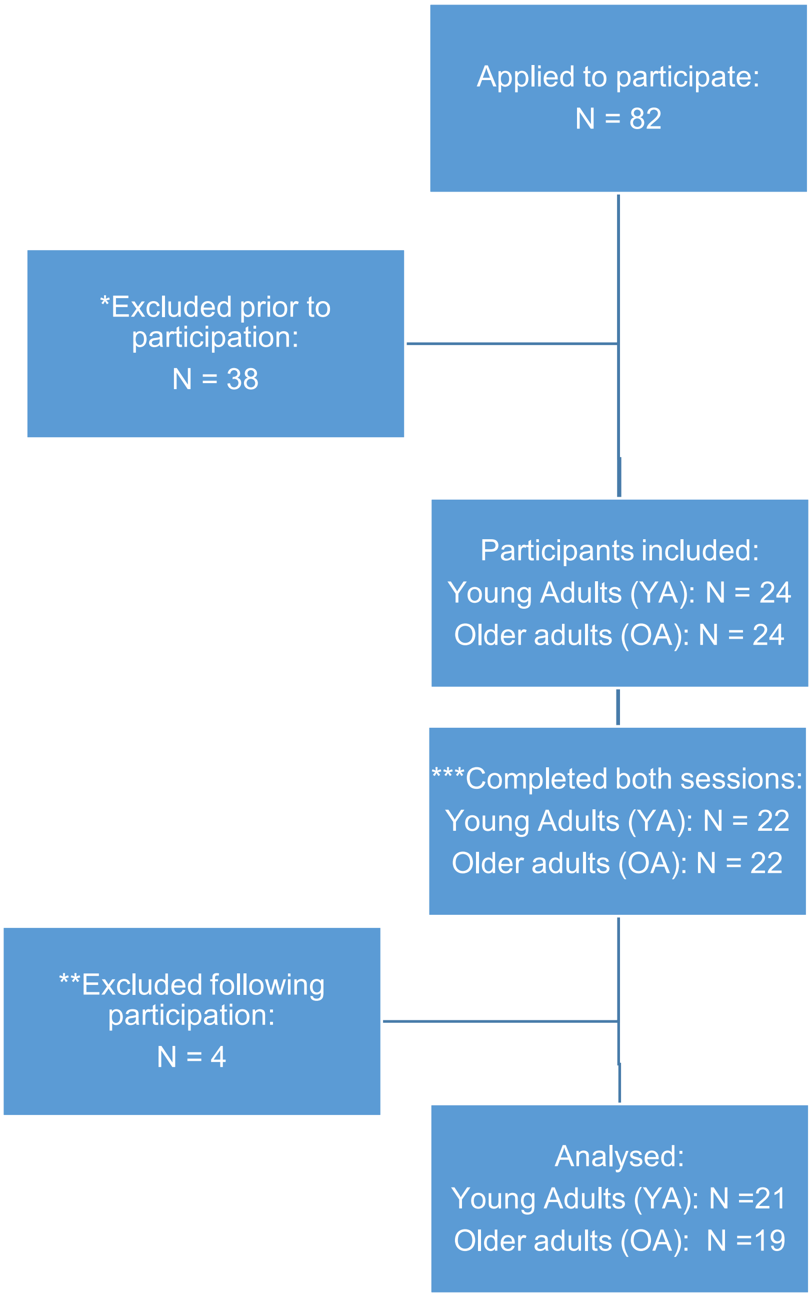

*REASONS FOR EXCLUSION:

Left-handed(n=1), retrained to be right-handed (n=2), adverse childhood experience (e.g. abuse) (n=7), mental health diagnosis (n=7), taking medications that affect central nervous system (n=11), neurological condition (n=9), medical condition (n=1).

**REASONS FOR EXCLUSION:

Poor cognitive task performance due to response confusion (e.g. pressed spacebar continuously) (n=4).

***ATTRITION:

4 participants did not attend session 2 because of enforced Covid19 lock-down (March 2020).

Study 2A Participant record (online only study, part of a wider project)

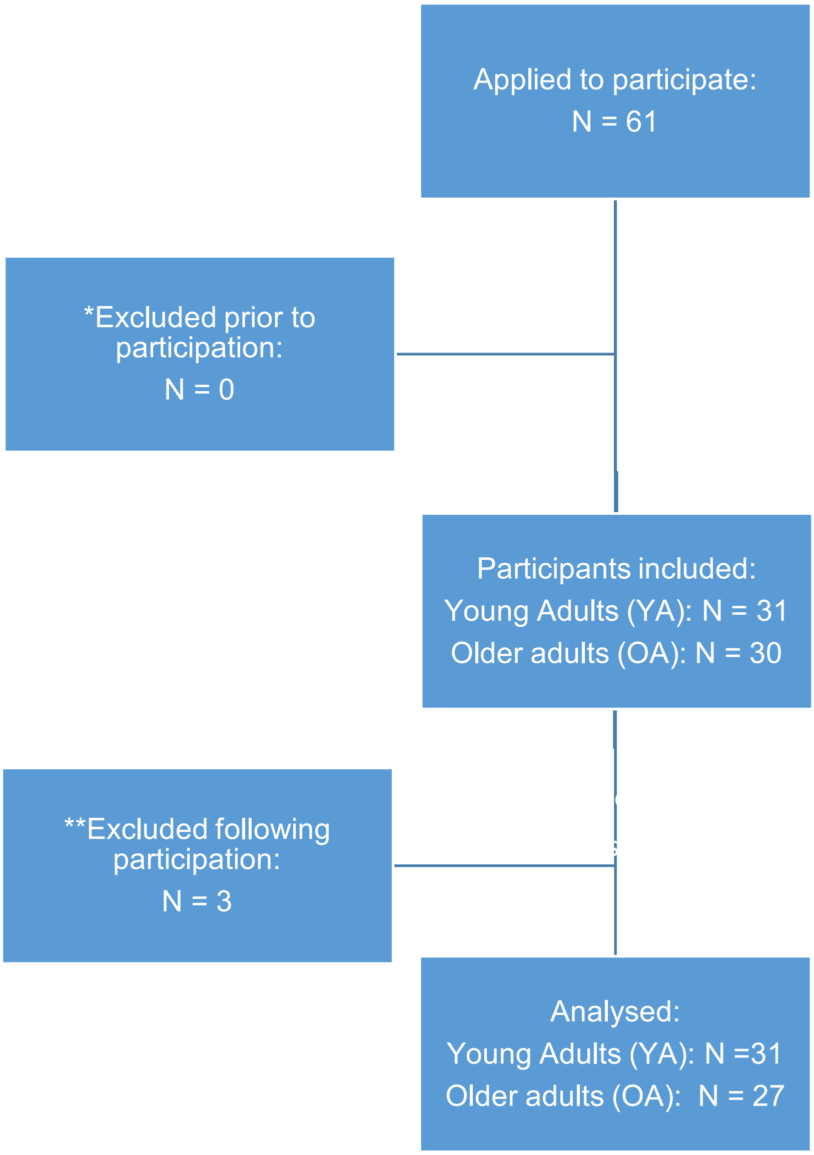

**<u>REASONS FOR EXCLUSION</u>:

Consumed alcohol within 12 hours of taking part in study (n=2); medications that cause drowsiness (n=1).

Study 2B Participant record (online only study, part of a wider project)

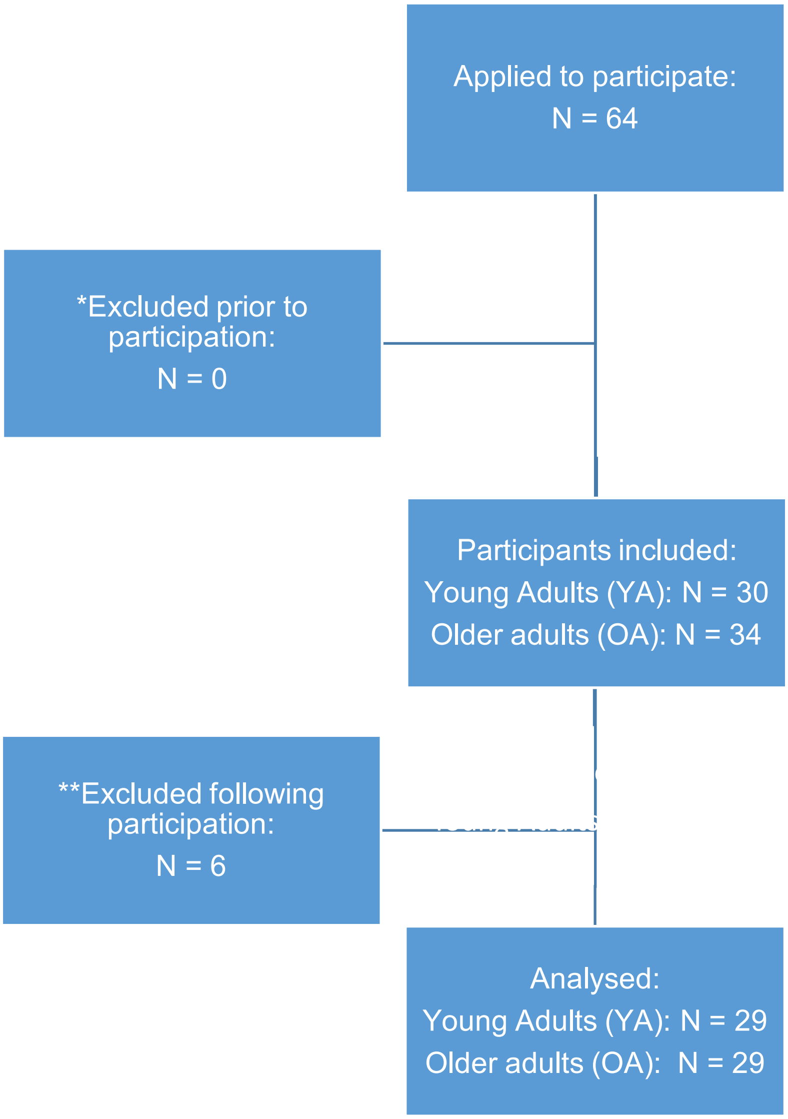

**<u>REASONS FOR EXCLUSION</u>:

Consumed alcohol within 12 hours of taking part in study (n=3); poor cognitive performance due to response confusion (n=3).
