# Supplementary material for: Do non-traumatic stressful life events and ageing negatively impact working memory performance and do they interact to further impair working memory performance?
Source: PLoS One. 2023 Nov 29;18(11):e0290635. doi: 10.1371/journal.pone.0290635 (PMC10686508; doi:10.1371/journal.pone.0290635)
Supplement: S2 Appendix — (PDF) [file pone.0290635.s007.pdf]

S3 Appendix 2. Additional tasks administered during the study (description and results)

Brief Resilience Scale

We wanted to extend Marshall's study protocol by adding resilience as a factor to enhance our understanding of how cumulative stress might affect cognition, given participants' ability to recover from stressful events. To this end, we administered the Brief Resilience Scale (BRS) [65]. The BRS has good internal consistency (Cronbach α <.95 > .70) and test-retest validity (interclass correlation coefficient .69 to .62) with a range of populations [e.g. 66, 67] and was found to be well-suited to stress-related contexts [68]. Participants were asked to self-report the extent to which they agreed with 6 statements on a scale of 1 ('Strongly Disagree') to 5 ('Strongly Agree'). Three of the statements were worded positively (items 1,3,5) and 3 negatively (items 2,4,6). Scores were derived by reverse-scoring items 2, 4 and 6 and then calculating the mean of all items. A higher mean score indicates greater resilience; previous research has shown that the BRS is negatively associated with physical symptoms and negative affect (e.g. irritability and distress) [65].

Subjective Sleep Quality

We also added the Pittsburgh Sleep Quality Index (PSQI) [69] to the study protocol to assess sleep quality, asking participants to report their sleep quality over the past month. Sleep quality has been consistently linked to variability in cognitive performance, stress, anxiety and illness [69-74]. Individual items in the PSQI yielded a Cronbach α of 0.83, indicating a high degree of internal consistency. Test-retest reliability revealed coefficient of .85 and there was good discriminant validity between clinical (depressed, disorders initiating and maintaining sleep, disorders of excessive somnolence) and control groups ($p < .001$). We used only questions 5 and 6 to keep the experiment short to reduce fatigue. Both questions were rated on a 4-point scale (score range: 0 to 3). Question 5a, in this study,

provided an index for ‘sleep latency’ and was rated as: ‘Not during the past month’ = 0 to ‘Three or more times a week’ = 3. Question 5 b-j comprises 10 questions assessing ‘sleep disturbances’ rated as per Q5a above. These values were summed for each participant. Summed totals were grouped into one of 4 brackets: 0; 1-9; 10-18; or 19-27, then recoded as a score of 0, 1, 2 or 3, respectively. Question 6 is a single question measuring ‘subjective sleep quality’ rated from ‘Very good’ = 0 to ‘Very bad’ = 3.

A global score, which had a score range of 0 – 9, was computed by summing the 3 aforementioned components, namely ‘sleep latency’, ‘sleep disturbances’ and ‘subjective sleep quality’. Note that these methods are adapted from the original PSQI which yields a global score of 0 – 21, based on 7 components.

Results

S3 Appendix Table 1. Descriptive statistics and p-values for self-reported resilience and sleep quality by age, by stress group for each study.

Brief Resilience Scale

	**Young Adults**		**Older Adults**		
	Low Stress[a] (n=11)	High Stress[a] (n=10)	Low Stress[a] (n=10)	High Stress[a] (n=9)	p
**Study 1 (N=40)**	3.58 (0.20)	3.87 (0.28)	3.90 (0.20)	3.80 (0.25)	≥0.401[b,c]
**Study 2A (N=58)**	3.33 (0.22)	3.61 (0.26)	3.76 (0.26)	3.27 (0.29)	≥0.242[b,c]
**Study 2B (N=58)**	2.99 (0.26)	3.18 (0.23)	3.87 (0.16)	3.76 (0.16)	≥0.562[b,c]

Sleep Quality (summed components range: 0 - 9)

	**Young Adults**		**Older Adults**		
	Low Stress[a] (n=11)	High Stress[a] (n=10)	Low Stress[a] (n=10)	High Stress[a] (n=9)	p
**Study 1 (N=40)**	3.18 (0.41)	2.6 (0.37)	2.4 (0.39)*	3.89 (0.46)*	≥0.025[b,c]
**Study 2A (N=58)**	2.25 (0.31)	2.87 (0.34)	2.5 (0.33)	3.54 (0.5)	≥0.091[b,c]
**Study 2B (N=58)**	2.60 (0.51)	3.14 (0.43)	2.53 (0.44)	3.50 (0.38)	≥0.116[b,c]

[a] Mean (SE). Standard error obtained via BCa Bootstrap with 1000 samples.

[b] Independent samples t-test (low vs high stress) were performed by age group.

[c] Additional Mann-Whitney U test were performed with similar outcomes.

* significant at < 0.05

** significant at < 0.01
