# Supplementary material for: Do non-traumatic stressful life events and ageing negatively impact working memory performance and do they interact to further impair working memory performance?
Source: PLoS One. 2023 Nov 29;18(11):e0290635. doi: 10.1371/journal.pone.0290635 (PMC10686508; doi:10.1371/journal.pone.0290635)
Supplement: S4 Appendix — (PDF) [file pone.0290635.s009.pdf]

S7 Appendix 4.

Study 1 Test Design and Procedure

Participants attended 2 sessions at least a week apart, at the same time of day. Participants received both sham and active stimulation. Order of treatment and task version was counter-balanced and randomised within age group. The Bayesian meta-analysis included session 1 data only. A univariate analysis of variance (ANOVA) was conducted on these data with age group, stress group and stimulation order as factors to evaluate the impact of the transcranial alternating current stimulation treatment on cognitive performance given that half of the participants received active stimulation in session 1 (YA_n=10;OA_n=9). The ANOVA results revealed a main effect for stress group only ($F(1, 32)$ 6.789, $p = 0.014$). All other main effects and interaction effects were not statistically significant (p's ≥ 0.106), indicating that stimulation did not have a significant impact on performance overall nor did it affect age or stress groups in a systematic way.

Session 1 only:

- Medical screening
- Procedure and nature of tasks briefly explained
- Informed consent
- All tasks to be administered fully explained
- Self-report measures (as detailed for Studies 2A and 2B overleaf)

Procedure for both sessions:

Pre-stimulation (offline)

Task	Duration
N-back practice (1-back, 2-back)	5 min
Spatial Mnemonic Discrimination Task – time 1	25 min
Head measurement and electrode placement	10 min
tACS comfort/phosphene assessment	30 s
Comfort Visual Analogue – time 1	30 s

During 20 min transcranial alternating current stimulation (online): 1500 µA (peak-to-peak)

Task	Duration
Comfort Visual Analogue – time 2	30 s
Watch nature video (habituation)	4 min 30 s
Picture Free Recall Task (encoding phase)	2 min
2-back task	7 min
Picture Free Recall Task (recall phase)	2 min
Comfort Visual Analogue – time 3	30 s

Post-stimulation (offline)

Task	Duration
Comfort Visual Analogue – time 4	30 s
Electrode removal	5 min
Spatial Mnemonic Discrimination Task – time 2	25 min

Session 2 only:

- On/off judgement for sessions 1 and 2

Study 2A and 2B Test Design and Procedure

Study 2A & 2B Test Design and Procedure

Participants signed up via the Prolific participant recruitment platform. Following self-assessment for eligibility they completed one test session, which was held in the morning for older participants and in the afternoon for young participants, which was roughly in line with time of day Study 1 participants participated. We also excluded for handedness and most of the same exclusion criteria to be consistent with the sample for Study 1.

Procedure

Study 2A and Study 2B

- Welcome screen
- Information Sheet
- Exclusion Criteria
- Informed Consent
- Biodemographic Information questionnaire
- Health and lifestyle questionnaire
- Life Events Questionnaire:
  - Life Events Scale for Students (LESS): 18-35 yrs
  - Social Readjustment Rating Scale (SRRS): 60 – 85 yrs
- Pittsburgh Sleep Quality Index
- Perceived Stress Scale (PSS-10)
- Brief Resilience Scale (BRS)
- STAI-S
- STAI-T

Cognitive Task: n-back

Study 2A

- 1-BACK Practice:
  - Block 1 [20 trials]
- 2-BACK Practice:
  - Block 1 [20 trials]
- 2-BACK Experimental Trials:
  - Block 1 [40 trials]
  - Block 2 [40 trials]
  - Block 3 [40 trials]

Study 2B

- 1-BACK Practice:
  - Block 1 [20 trials]
- 1-BACK Experimental Trials:
  - Block 1 [40 trials]
  - Block 2 [40 trials]
  - Block 3 [40 trials]
- 2-BACK Practice:
  - Block 1 [20 trials]
- 2-BACK Experimental Trials:
  - Block 1 [40 trials]
  - Block 2 [40 trials]
  - Block 3 [40 trials]
