# Supplementary material for: Do non-traumatic stressful life events and ageing negatively impact working memory performance and do they interact to further impair working memory performance?
Source: PLoS One. 2023 Nov 29;18(11):e0290635. doi: 10.1371/journal.pone.0290635 (PMC10686508; doi:10.1371/journal.pone.0290635)
Supplement: Attachment — Submitted filename: Response to reviewer.docx [file pone.0290635.s011.docx]

Dr. Amir-Homayoun Javadi

Editor,

Plos One

**MS # PONE-D-22-18670:** **Do non-traumatic stressful life events and ageing negatively impact working memory performance and do they interact to further impair working memory performance? (Wallace and colleagues)**

Dear Dr. Javadi,

We would like to thank you for your insightful comments and suggestions. Our replies are set out point by point below:

**Reviewer Point 1**: We…encourage you to add the below analysis and resubmit. 

Currently the statistical power of the report relies on the number of participants. While this might be an accurate comparison, I would encourage you to run an appropriate power calculation to further support your claims. Please see this paper as an example for reporting of null results:

Smittenaar, P., Prichard, G., FitzGerald, T. H. B., Diedrichsen, J., & Dolan, R. J. (2014). Transcranial Direct Current Stimulation of Right Dorsolateral Prefrontal Cortex Does Not Affect Model-Based or Model-Free Reinforcement Learning in Humans. PLOS ONE, 9(1), e86850. [https://doi.org/10.1371/journal.pone.0086850](https://linkprotect.cudasvc.com/url?a=https%3a%2f%2fdoi.org%2f10.1371%2fjournal.pone.0086850&c=E,1,UiZ4o-IQRmS0YI8W-93OEB9b1okpWI3-YmIX84t5wkKZ3suAO7oI9VDo1nt57q9DdHaUhIhWR-o5q6E1RblZ_4j04CRh23o5ayTZhTgwSLk22glwH7mQ&typo=1)

**Reply to Point 1:** We appreciated the suggestion to follow the approach taken by Smittenaar, P., Prichard, G., FitzGerald, T. H. B., Diedrichsen, J., & Dolan, R. J. (2014). In reviewing their methods, we noted that their rationale for using a model comparison to test the robustness of their null result was to mimic the approach of Bayesian methods which provides evidence for either H0 or H1 (or neither). On the contrary, our intention was to use a Bayesian approach to avoid the ambiguity that sometimes accompanies p-values. Thus, for the main analysis we have simplified the manuscript by removing the univariate ANOVA analyses and reported the outcomes of our Bayesian meta-analysis. We have now provided a frequentist meta-analysis, including power analysis, in the supplemental files for interested readers. Please refer to S5_Appendix3.

**Reviewer Point 2**: In addition, you can also run power calculation suitable for your analyses.

**Reply to Point 2:** We have provided a robustness check for our Bayesian analysis to support our results. Adjustments can be found in the manuscript (line 323, page 15 under ‘methods’ and 399, page 18 under ‘results’).

We hope that the manuscript is clearer in its focus and we look forward to hearing from you.

Best regards,

Denise Wallace

On behalf of the authors
